# Correction to “Pros and cons for statins use and risk of Parkinson’s disease: An updated perspective”

**DOI:** 10.1002/prp2.1221

**Published:** 2024-06-06

Al‐kuraishy HM , Al‐Gareeb AI , Alexiou A , et al. Pros and cons for statins use and risk of Parkinson‘s disease: An updated perspective. Pharmacol Res Perspect. 2023; 11:e01063. 10.1002/prp2.1063 36811160 PMC9944858

In Introduction section, the sentence “Remarkably, the incidence of PD in the general population is 0.3% and reaches 4% above the age of 80 years.^6^” is incorrect. The sentence should have read: “Remarkably, the incidence of PD in the general population in 2019 was 0.001%, with an increase of 100% since 2000, and the population prevalence of PD increases from about 1% at age 60 to 4% by age 80.^6,76,77^”. Reference 76 is: The WHO's Parkinson disease technical brief, 2022, Retrieved from https://www.who.int/news/item/14‐06‐2022‐launch‐of‐who‐s‐parkinson‐disease‐technical‐brief and Reference 77 is: Centers for Disease Control and Prevention (CDC), Retrieved from https://www.cdc.gov/genomics/hugenet/casestudy/parkinson/parkcoffeecase.htm].

We apologize for this error.

